# *How* does different types of exercise promote cognitive function? What do we know?

**DOI:** 10.1002/alz.088041

**Published:** 2025-01-09

**Authors:** Teresa Liu‐Ambrose

**Affiliations:** ^1^ Vancouver Coastal Health Research Institute, Vancouver, BC, Canada; University of British Columbia, Vancouver, BC, Canada; Centre for Aging SMART, Vancouver Coastal Health Research Institute, Vancouver, BC, Canada; Djavad Mowafaghian Centre for Brain Health, Vancouver, BC Canada

## Abstract

Our current understanding of how exercise promotes cognitive function largely stems from animal studies and is restricted to aerobic exercise training (AT). It is widely hypothesized that AT induces neurotrophic factor cascades (i.e., BDNF, IGF‐1, and VEGF), a central mechanism mediating exercise‐dependent benefits in cognitive performance. However, evidence regarding the effect of AT on neurotrophic factors in humans is equivocal. Notably, whether AT‐induced changes in neurotrophic factors are associated with AT‐induced changes in cognitive function is largely unknown. The dearth of mechanistic evidence is even more pronounced for resistance training (RT).

Both AT and RT may also promote cognitive function by reducing peripheral cardiometabolic risk factors for neurodegeneration (e.g., hypertension, type 2 diabetes, hypercholesterolemia), as well as systemic inflammation associated with these risk factors. Specifically, cardiovascular disease and type 2 diabetes are associated with chronic low‐grade systemic inflammation reflected by elevated levels of C reactive protein and cytokines. Systemic inflammation can impair neurotrophic factor signaling, exacerbate the metabolic syndrome, and accelerate cognitive decline. Similar to neurotrophic factors, the roles of cardiometabolic risk and systemic inflammation in exercise‐induced improvements in human cognitive performance are not well established.

A better understanding of the underlying biological mechanisms of different types of exercise training will greatly advance our ability to refine and develop novel exercise (and other) strategies for dementia prevention. In this session, we will discuss/debate the selection and measurement of biomarkers in randomized controlled trials of exercise in individuals with or without cognitive impairment.

